# Interpretable Prediction and Analysis of PVA Hydrogel Mechanical Behavior Using Machine Learning

**DOI:** 10.3390/gels11070550

**Published:** 2025-07-16

**Authors:** Liying Xu, Siqi Liu, Anqi Lin, Zichuan Su, Daxin Liang

**Affiliations:** 1 School of Food Engineering, Harbin University, Harbin 150086, China; 2 Key Laboratory of Bio-Based Material Science and Technology (Ministry of Education), Northeast Forestry University, Harbin 150040, China; 3 College of Chemistry and Chemical Engineering, China University of Petroleum (East China), Qingdao 266580, China

**Keywords:** PVA hydrogel, interpretable machine learning, SHAP analysis, feature importance, hyperparameter optimization

## Abstract

Polyvinyl alcohol (PVA) hydrogels have emerged as versatile materials due to their exceptional biocompatibility and tunable mechanical properties, showing great promise for flexible sensors, smart wound dressings, and tissue engineering applications. However, rational design remains challenging due to complex structure–property relationships involving multiple formulation parameters. This study presents an interpretable machine learning framework for predicting PVA hydrogel tensile strain properties with emphasis on mechanistic understanding, based on a comprehensive dataset of 350 data points collected from a systematic literature review. XGBoost demonstrated superior performance after Optuna-based optimization, achieving R^2^ values of 0.964 for training and 0.801 for testing. SHAP analysis provided unprecedented mechanistic insights, revealing that PVA molecular weight dominates mechanical performance (SHAP importance: 84.94) through chain entanglement and crystallization mechanisms, followed by degree of hydrolysis (72.46) and cross-linking parameters. The interpretability analysis identified optimal parameter ranges and critical feature interactions, elucidating complex non-linear relationships and reinforcement mechanisms. By addressing the “black box” limitation of machine learning, this approach enables rational design strategies and mechanistic understanding for next-generation multifunctional hydrogels.

## 1. Introduction

Hydrogels, three-dimensional cross-linked polymer networks capable of absorbing substantial water while maintaining structural integrity, have emerged as versatile materials across biomedical, electronic, and environmental domains [1,2]. Among various systems, PVA hydrogels have attracted considerable attention due to their exceptional biocompatibility, tunable mechanical properties, and chemical stability [3]. The unique combination of hydrogen bonding networks and crystalline regions enables physically cross-linked formation without toxic chemical cross-linkers [4,5,6], allowing mechanical properties ranging from soft, tissue-like materials to robust, load-bearing structures [7]. This tunability positions PVA hydrogels as promising candidates for flexible sensors, smart wound dressings, drug delivery systems, and tissue engineering scaffolds [8,9,10].

Recent advances have demonstrated the remarkable versatility of PVA hydrogels across diverse technological applications. PVA-based conductive hydrogels have been successfully employed in human–machine interaction systems for intelligent rehabilitation training, leveraging their excellent sensing capabilities and biocompatibility [11]. Self-healing ionic conductive PVA hydrogels have shown exceptional performance in wearable sensor applications, offering multi-sensing capabilities while maintaining transparency and mechanical robustness [12]. Additionally, PVA hydrogels have been integrated into sophisticated sensing platforms, such as SERS sensors for antitumor drug detection and robust conductive systems based on hydrotalcite nanocomposites [13]. These applications highlight the potential of PVA hydrogels to address complex technological challenges across healthcare, environmental monitoring, and biomedical diagnostics. However, the full realization of PVA hydrogels’ potential in these applications depends critically on precise control and understanding of their mechanical properties and modulus characteristics. A comprehensive investigation of the structure–property relationships governing PVA hydrogel mechanics would significantly contribute to advancing these emerging fields and enabling rational design of next-generation hydrogel-based devices [14,15,16].

Machine learning (ML) approaches have demonstrated remarkable success in materials science for predicting complex material properties and understanding structure–property relationships [17]. SHAP analysis has emerged as a powerful interpretability technique to address the “black box” problem in ML models, enabling researchers to gain mechanistic insights into feature contributions and underlying material behavior. However, comprehensive ML studies focusing specifically on PVA hydrogel mechanical property prediction remain limited [18].

To address these limitations, this study presents a machine learning framework for predicting PVA hydrogel tensile strain properties based on key formulation and processing parameters including PVA molecular weight, degree of hydrolysis, concentration, freeze–thaw cycles, cross-linking methods, and conductive content. Through systematic literature screening of 46 research papers and collection of 350 data points, we developed an Extreme Gradient Boosting (XGBoost) model and employed Shapley Additive Explanations (SHAP) analysis to elucidate mechanistic contributions of individual features. The analysis revealed that PVA molecular weight dominates mechanical performance, followed by degree of hydrolysis and cross-linking parameters, while feature interactions significantly influence optimal parameter ranges. This work establishes an interpretable framework for rational design of these materials.

## 2. Results and Discussion

### 2.1. Analysis of Input Features Prior to Model Development

In this study, a comprehensive dataset was assembled encompassing key formulation and processing parameters that influence PVA hydrogel mechanical properties. The continuous variables include PVA molecular weight (PVA_MW), degree of hydrolysis (PVA_DH), PVA concentration (PVA_Conc), freeze–thaw cycles (FC), cross-linking method (CLM), and conductive content (CC). Additionally, categorical variables were incorporated for chemical cross-linkers (Borax, CaCl_2_, EG, GL, MBA, PBA) and conductive types (AgNPs, CNF, CNT, Ionic_only, LDH, MXene, PANI, PEDOT:PSS, TA, rGO, PPy), which were subsequently encoded as binary variables for machine learning analysis. The target variable is tensile strain (Tensile_Strain).

**Figure 1:**
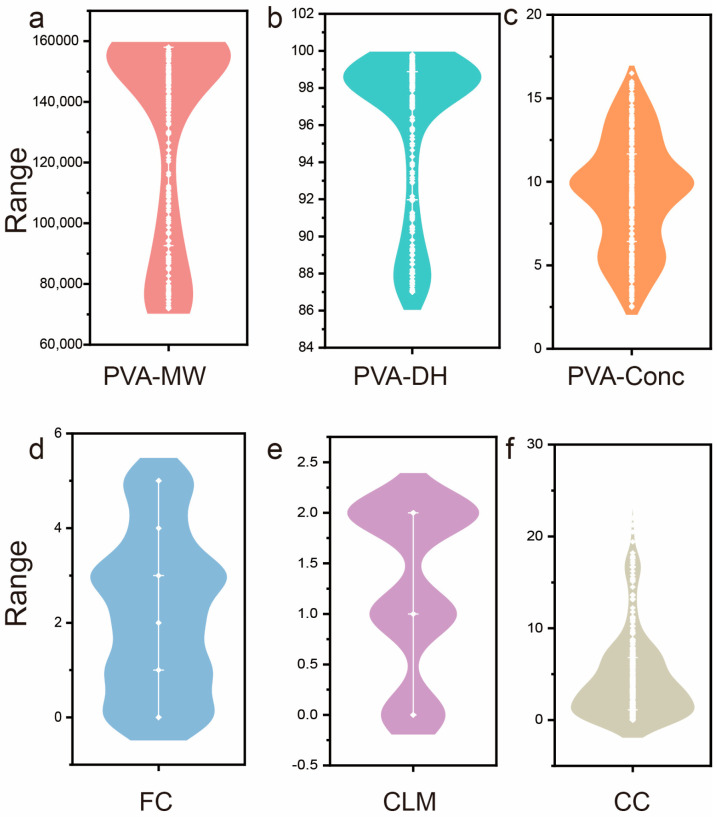
Distribution analysis of continuous input features. (**a**) PVA_MW, (**b**) PVA_DH, (**c**) PVA_Conc, (**d**) FC, (**e**) CLM, (**f**) CC.

The distribution analysis of continuous features reveals important characteristics of the assembled dataset (Figure 1). PVA_MW shows a broad distribution from 70,000 to 160,000 Da, with a concentration around 100,000–120,000 Da (Figure 1a), while PVA_DH exhibits a narrow distribution centered at 98–99% (Figure 1b), indicating high hydrolysis degrees across samples. PVA_Conc demonstrates a multimodal distribution ranging from 2% to 18% w/w, with distinct peaks at lower and higher concentrations (Figure 1c). FC displays relatively uniform distribution across 0–5 cycles (Figure 1d), CLM shows bimodal distribution indicating different cross-linking strategies (Figure 1e), and CC exhibits a highly skewed distribution with most samples containing low conductive content (0–5%) and fewer samples with higher loadings up to 20% (Figure 1f). The diverse distribution patterns observed across all features demonstrate the comprehensive nature of the dataset, encompassing a wide range of formulation parameters that are representative of practical PVA hydrogel synthesis conditions. These distribution characteristics provide a solid foundation for robust machine learning model training and ensure that the developed predictive framework can capture the complex relationships between processing parameters and mechanical properties across the entire experimental space [18,19,20].

**Figure 2:**
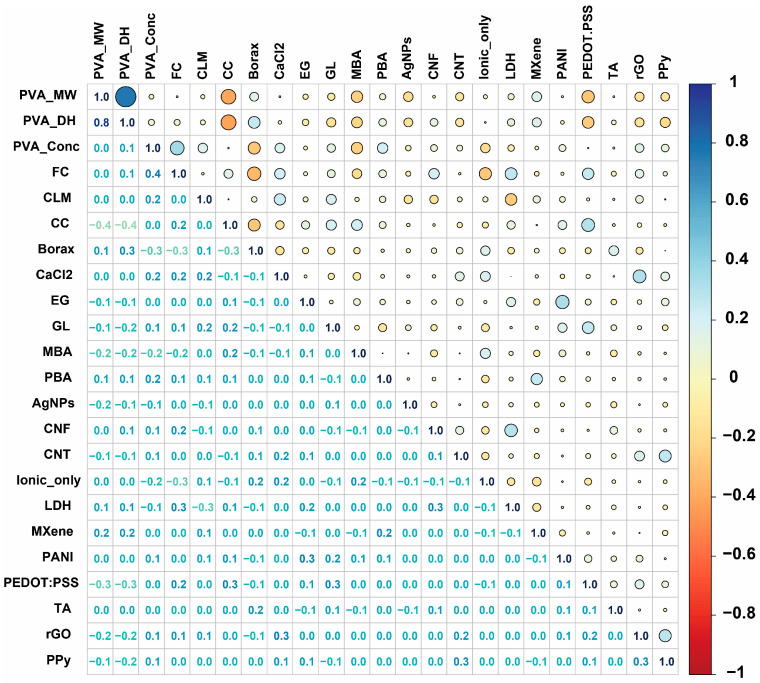
Pearson correlation matrix of input features.

The Pearson correlation matrix reveals important relationships between input features that inform feature selection and model interpretation (Figure 2). Among the continuous variables, most features exhibit relatively weak correlations with each other, indicating good feature independence. PVA_MW shows minimal correlation with other continuous parameters, while PVA_DH demonstrates slight positive correlations with several chemical cross-linkers. PVA_Conc exhibits weak correlations with most features, suggesting its independent contribution to hydrogel properties [21,22]. FC shows moderate correlations with certain conductive materials, particularly with some nanomaterials, indicating potential interactions between freeze–thaw processing and filler incorporation [23,24,25].

The categorical features representing chemical cross-linkers and conductive types generally show weak to moderate correlations among themselves, which is expected given their binary encoding nature [4,7,26]. Notable correlations exist between certain conductive materials, such as moderate positive correlations among some carbon-based materials (CNF, CNT, rGO) and conducting polymers (PANI, PEDOT:PSS). Most correlation coefficients remain below 0.5, indicating an absence of severe multicollinearity issues that could compromise model performance [27,28,29,30]. The relatively low inter-feature correlations suggest that each parameter contributes unique information to the prediction model, justifying the inclusion of all features in the machine learning framework [13,31].

### 2.2. Model Development

**Figure 3:**
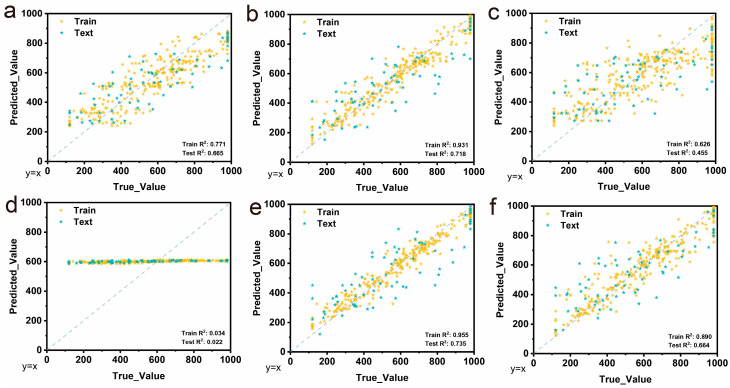
Machine learning model performance comparison for tensile strain prediction. (**a**) AdaBoost, (**b**) GBR, (**c**) KNN, (**d**) SVM, (**e**) XGBoost, (**f**) MLP.

To establish an effective predictive framework for PVA hydrogel tensile strain properties, six different machine learning algorithms were evaluated and compared: AdaBoost, Gradient Boosting Regression (GBR), K-Nearest Neighbors (KNN), Support Vector Machine (SVM), Extreme Gradient Boosting (XGBoost), and Multi-Layer Perceptron (MLP). Figure 3 presents the predicted versus true value plots for all models, providing comprehensive performance evaluation across both training and testing datasets.

The performance analysis reveals significant variations among different algorithms. AdaBoost demonstrates moderate predictive capability with a training R^2^ of 0.771 and test R^2^ of 0.665 (Figure 3a), indicating reasonable model performance but with notable generalization gap. GBR exhibits exceptional training performance with R^2^ of 0.931 but shows substantial overfitting with test R^2^ dropping to 0.718 (Figure 3b), suggesting limited generalizability to unseen data. KNN displays poor overall performance with extremely low training R^2^ of 0.034 and test R^2^ of 0.022 (Figure 3c), with predictions clustered around a constant value near 600, indicating the algorithm’s inability to capture the underlying data patterns effectively [32,33].

SVM shows promising results with a high training R^2^ of 0.955 and reasonable test performance R^2^ of 0.735 (Figure 3d), demonstrating good predictive capability but with some overfitting tendency. MLP achieves excellent training performance with an R^2^ of 0.890 and maintains good generalization with a test R^2^ of 0.664 (Figure 3e), showing balanced performance across both datasets. Most notably, XGBoost delivers outstanding performance, with the highest training R^2^ of 0.955 and a superior test R^2^ of 0.735 (Figure 3f), demonstrating both excellent predictive accuracy and robust generalization capability [34,35].

The comparative analysis clearly indicates that XGBoost outperforms other algorithms in terms of both accuracy and generalization ability. Unlike GBR which shows severe overfitting, XGBoost maintains consistent performance between training and testing phases, suggesting better model stability. The superior performance of XGBoost can be attributed to its advanced ensemble learning mechanism, effective handling of feature interactions, and built-in regularization techniques that prevent overfitting. Additionally, XGBoost’s inherent feature importance calculation and compatibility with SHAP analysis make it an ideal choice for subsequent mechanistic interpretation studies [36]. Based on these comprehensive performance evaluations, XGBoost was selected as the optimal model for further optimization and detailed analysis of PVA hydrogel property prediction.

### 2.3. Optimized XGBoost Model Performance and Feature Importance Analysis

**Figure 4:**
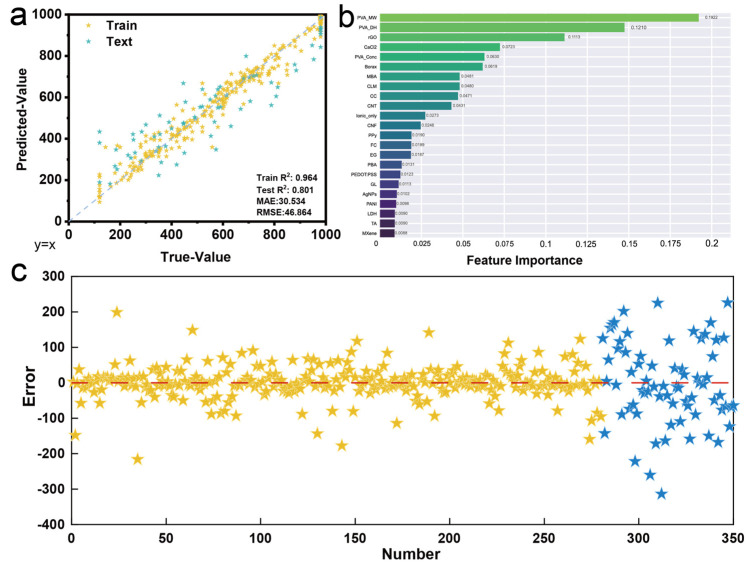
(**a**) Predicted vs. true values with performance metrics, (**b**) feature importance ranking, (**c**) residual analysis for training and test datasets.

Following the initial model comparison, the XGBoost algorithm was subjected to systematic hyperparameter optimization using the Optuna framework to maximize predictive performance. The optimized XGBoost model demonstrates exceptional performance, with significantly improved metrics compared to the preliminary version (Figure 4a). The training R^2^ increased to 0.964 while maintaining excellent generalization capability with a test R^2^ of 0.801, representing a substantial improvement over the initial XGBoost performance. The model achieves low prediction errors with MAE of 30.534 and RMSE of 46.864, indicating high accuracy in tensile strain prediction across the entire range of experimental values. The strong alignment between predicted and true values for both training and testing datasets confirms the model’s robustness and reliability for practical applications [37,38].

The feature importance analysis reveals critical insights into the relative contributions of different parameters to PVA hydrogel tensile strain properties (Figure 4b). PVA_MW emerges as the most influential feature with an importance score of approximately 0.19, highlighting the fundamental role of molecular weight in determining mechanical properties through polymer chain entanglement and crystallization behavior [13,22,39]. rGO follows as the second most important feature with a score of ~0.11, indicating the significant impact of reduced graphene oxide incorporation on hydrogel mechanics [40,41,42]. CaCl_2_ shows notable importance (~0.07), reflecting the crucial role of ionic cross-linking in modulating mechanical properties. PVA_Conc demonstrates moderate importance (~0.06), confirming the expected influence of polymer concentration on network density and mechanical performance [26,29,43]. Interestingly, several conductive materials including Borax, MBA, and various nanomaterials show relatively lower but still meaningful contributions, suggesting their secondary but important roles in fine-tuning mechanical properties. The residual analysis demonstrates overall model reliability with generally random scatter around zero for most data points (Figure 4c). Training data exhibit uniform residual distribution within ±100 units. The residual analysis reveals some data points that deviate from the ideal zero-residual line, which can be attributed to several factors. Experimental variations across different research groups, including differences in testing protocols and sample preparation methods, contribute to prediction deviations. Additionally, variations in measurement techniques and equipment precision across literature sources, as well as inherent material property differences from various PVA suppliers, influence the residual distribution. These factors highlight the challenges of developing predictive models from literature-based datasets and suggest areas for future model refinement through more standardized experimental protocols.

### 2.4. Feature Importance and Interpretability Analysis

To gain a deeper mechanistic understanding of the XGBoost model predictions and elucidate the underlying structure–property relationships, SHAP analysis was employed to quantify individual feature contributions to tensile strain predictions. The SHAP analysis provides both global feature importance and local explanations for individual predictions [17,19], offering unprecedented insights into PVA hydrogel mechanical behavior.

**Figure 5:**
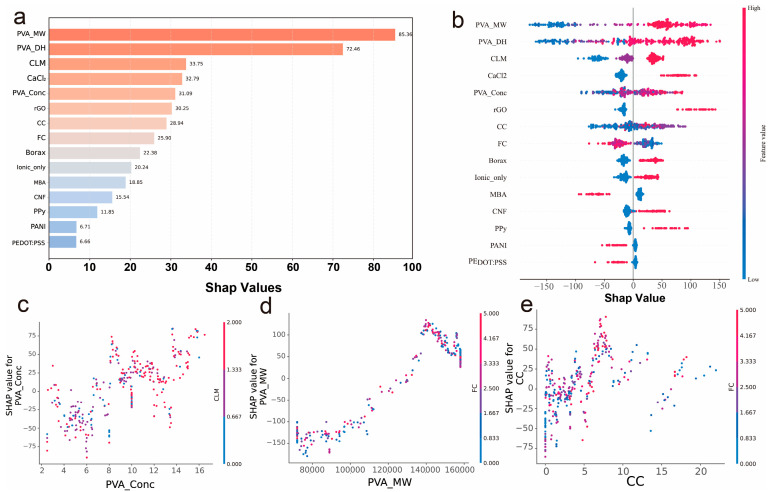
(**a**) Global SHAP feature importance values, (**b**) SHAP summary plot showing individual feature contributions across all samples, (**c**–**e**) SHAP dependence plots for PVA_Conc, PVA_MW, and CC, respectively, with color coding indicating interaction effects.

The global SHAP importance analysis reveals the hierarchical contributions of features to tensile strain properties, providing critical insights for rational hydrogel design (Figure 5a). SHAP values represent quantitative measures of feature importance, where higher values indicate greater contribution to model predictions. PVA_MW emerges as the dominant factor with a SHAP value of 84.94, indicating that molecular weight accounts for the largest proportion of predictive influence among all features, fundamentally governing mechanical performance through polymer chain entanglement dynamics and crystalline domain formation [12,31,44]. This finding suggests that molecular weight selection should be the primary consideration in hydrogel formulation, with higher molecular weights promoting enhanced chain entanglement density and load transfer efficiency. PVA_DH demonstrates substantial importance (72.46), directly influencing hydrogen bonding capacity and crystallization tendency higher DH values facilitate stronger intermolecular interactions and more organized crystalline structures, leading to improved mechanical integrity [45,46,47]. The significant contributions of CLM (33.75) and CaCl_2_ (32.78) highlight the critical role of cross-linking strategies, where ionic cross-linking through Ca^2+^ ions creates additional physical junction points that enhance network connectivity and stress distribution [24,48,49]. Notably, rGO shows considerable importance (30.25), indicating that graphene incorporation provides effective reinforcement through strong interfacial interactions and load transfer mechanisms [22,31,39].

The SHAP summary plot provides mechanistic insights into how each feature influences tensile strain across different value ranges (Figure 5b). PVA_MW exhibits predominantly positive SHAP values for higher molecular weights (red regions), confirming that increased chain length enhances mechanical performance through greater entanglement density and improved stress transfer pathways [12,31,44]. The distribution pattern suggests an optimal molecular weight threshold above which mechanical benefits become pronounced, guiding formulation strategies toward higher MW PVA selection. PVA_DH shows a more complex pattern with both positive and negative contributions, indicating that while high hydrolysis degrees generally promote hydrogen bonding, excessive hydrolysis may lead to increased crystallinity that could compromise flexibility [45,46,47]. This suggests an optimal DH range that balances hydrogen bonding strength with network flexibility. The binary cross-linking features (CLM, CaCl_2_) display clear positive contributions when present, emphasizing the necessity of appropriate cross-linking for mechanical enhancement [24,48,49]. Importantly, the rGO distribution shows strong positive SHAP values when present, but also reveals that not all graphene incorporations yield equivalent benefits, suggesting that processing conditions and dispersion quality critically influence reinforcement effectiveness [22,31,39]. For conductive additives, the varied SHAP distributions indicate that material selection and loading optimization are crucial for achieving desired mechanical properties while maintaining conductivity. Following the primary features, the secondary parameters reveal important fine-tuning mechanisms for PVA hydrogel optimization. Borax and MBA demonstrate consistent positive SHAP contributions when present, confirming their effectiveness as cross-linking agents that enhance network connectivity through borate complexation and covalent bonding, respectively [24,50]. The freeze–thaw cycles (FC) show moderate positive effects, indicating that controlled crystallization during freeze–thaw processing creates beneficial physical cross-links [25,51]. Among the conductive materials, CNT and CNF exhibit predominantly positive SHAP values, suggesting that these carbon-based nanofillers provide dual benefits of electrical conductivity and mechanical reinforcement. However, the conducting polymers (PANI, PEDOT:PSS, PPy) display more variable SHAP distributions [30,42,52], indicating that their mechanical contributions are highly dependent on processing conditions and dispersion quality, emphasizing the need for careful optimization when incorporating these materials for multifunctional applications.

The SHAP dependence plots reveal complex non-linear relationships and feature interactions for key parameters (Figure 5c–e). The PVA_Conc dependence plot (Figure 5c) demonstrates a complex relationship where moderate concentrations (6–10%) show predominantly positive SHAP values, while both very low and very high concentrations tend to have neutral or negative effects. This reflects optimal chain entanglement density at moderate concentrations, where sufficient polymer chains enable effective cross-linking without chain crowding effects that hinder molecular mobility. The color coding by CLM reveals important interaction effects, suggesting that the optimal concentration range depends significantly on the cross-linking method employed [25,49]. The PVA_MW dependence plot (Figure 5d) shows a generally positive correlation between molecular weight and SHAP values, with the strongest positive effects occurring at molecular weights above 120,000 Da. This threshold corresponds to the critical molecular weight for chain entanglement in PVA, above which enhanced mechanical properties result from increased entanglement density and crystallization potential. Interestingly, the interaction with FC reveals that the beneficial effect of high molecular weight is enhanced when combined with appropriate freeze–thaw cycling [5,41]. The CC dependence plot (Figure 5e) exhibits a non-monotonic relationship where low to moderate conductive content (2–8%) provides positive contributions to tensile strain, while higher loadings show diminishing or negative effects, likely due to filler agglomeration and network disruption [25,53].

These SHAP analyses collectively provide mechanistic insights into PVA hydrogel design principles. For enhanced mechanical performance, we recommend prioritizing high molecular weight PVA above 120,000 Da combined with optimized freeze–thaw cycles of 2–4 cycles to maximize chain entanglement and crystallization benefits, maintaining moderate PVA concentrations between 6 and 10% with appropriate ionic cross-linking agents such as CaCl_2_ for optimal network connectivity, and incorporating low-to-moderate conductive filler loadings of 2–8% to achieve reinforcement without network disruption. The dominant influence of PVA_MW and PVA_DH confirms the fundamental importance of polymer characteristics, while the significant contributions of cross-linking parameters and conductive additives highlight the critical role of formulation optimization. The identified parameter interactions suggest that synergistic combinations, particularly high MW PVA with controlled freeze–thaw processing and moderate filler loading with ionic cross-linking, can achieve superior mechanical properties compared to individual parameter optimization. The revealed interaction effects between features emphasize the need for holistic design approaches rather than individual parameter optimization, providing valuable guidance for rational hydrogel design and property tailoring, future experimental validation of these design principles would benefit from advanced characterization techniques such as MALLS (multi-angle laser light scattering) for precise molecular weight determination and conformational analysis, as demonstrated by Macarie and Pekar for vinyl-containing polymers, which could provide deeper insights into the structure–property relationships governing these synergistic effects [54].

## 3. Conclusions

This study successfully developed an interpretable machine learning framework for predicting PVA hydrogel tensile strain properties. XGBoost emerged as the optimal model after comprehensive evaluation, achieving exceptional performance with R^2^ values of 0.964 for training and 0.801 for testing datasets following Optuna-based hyperparameter optimization. The low prediction errors (MAE: 30.534, RMSE: 46.864) demonstrate reliable predictive capability for practical applications.

SHAP analysis revealed critical design principles governing PVA hydrogel mechanical behavior. SHAP importance values quantify individual feature contributions to model predictions, with higher values indicating greater influence on the target property. PVA molecular weight emerged as the dominant factor (SHAP importance: 84.94), followed by the degree of hydrolysis (72.46) and cross-linking parameters, emphasizing the fundamental roles of polymer chain characteristics and structural connectivity. The significant impact of rGO incorporation demonstrates effective graphene-based reinforcement strategies [40,48,55]. The identified optimal parameter ranges and revealed feature interactions provide valuable guidance for rational hydrogel design, emphasizing holistic optimization approaches [36,37].

The interpretable machine learning framework established here advances both fundamental understanding and practical applications of PVA hydrogels. This work represents the first comprehensive application of SHAP analysis to elucidate structure–property relationships in PVA hydrogels, providing quantitative mechanistic insights that were previously inaccessible through conventional analytical methods. By bridging complex formulation parameters with mechanical properties, this approach enables accelerated materials discovery and rational design strategies that surpass conventional design paradigms by providing predictive capability combined with mechanistic understanding. The novel integration of feature importance analysis with polymer physics principles establishes a new paradigm for interpretable materials design, addressing the critical gap between machine learning predictions and mechanistic understanding in soft materials research. Future advancement of predictive models would benefit from more comprehensive molecular characterization, including polydispersity indices, conformational analysis through GPC-MALLS, and detailed molecular weight distribution data, enabling higher-quality datasets and more accurate data-driven experimental design strategies. The methodology can be extended to other hydrogel systems and property predictions, potentially transforming advanced soft materials development for flexible electronics, biomedical devices, and smart material applications.

## 4. Materials and Methods

### 4.1. Data Collection and Preprocessing

This study utilized Web of Science as the primary data source, employing ‘PVA hydrogel’, ‘polyvinyl alcohol hydrogel mechanical properties’, and ‘PVA hydrogel tensile strain’ as search keywords to systematically collect experimental data from peer-reviewed literature published between 2017 and 2024. After comprehensive screening and quality assessment, 46 research papers were selected that provided complete formulation parameters and mechanical testing data for PVA hydrogels [3,4,5,6,7,8,9,10,11,12,13,21,22,23,24,25,26,27,28,29,30,31,39,40,41,42,43,44,45,46,47,48,49,50,51,52,53,55,56,57,58,59,60,61,62,63].

The collected data encompassed six continuous variables: PVA molecular weight (PVA_MW), degree of hydrolysis (PVA_DH), PVA concentration (PVA_Conc), freeze–thaw cycles (FC), cross-linking method (CLM), and conductive content (CC). Chemical cross-linkers were categorized into six types: Borax, CaCl_2_, ethylene glycol (EG), glutaraldehyde (GL), N,N′-methylenebisacrylamide (MBA), and polyboronic acid (PBA). Conductive materials were classified into eleven categories: silver nanoparticles (AgNPs), carbon nanofibers (CNF), carbon nanotubes (CNT), ionic conductors only (Ionic_only), layered double hydroxide (LDH), MXene, polyaniline (PANI), poly(3,4-ethylenedioxythiophene):poly(styrenesulfonate) (PEDOT:PSS), tannic acid (TA), reduced graphene oxide (rGO), and polypyrrole (PPy). One-hot encoding was applied to all categorical features to create binary numerical representations for machine learning analysis. Missing values were systematically handled using domain-specific approaches. For PVA_MW and PVA_DH parameters, when papers reported only one value, the missing parameter was estimated using established the molecular weight-degree of hydrolysis relationships from manufacturer specifications or the same research group’s previous work. For PVA_Conc, when not explicitly reported, concentrations were calculated from solid content percentages, polymer-to-solvent ratios, or preparation protocols described in experimental sections. The final dataset incorporated 23 input features.

### 4.2. Model Development and Evaluation Methodology

The dataset was split into training and testing sets using an 80:20 ratio to evaluate model generalization performance. Six machine learning algorithms were systematically evaluated: AdaBoost (ADB), Gradient Boosting Regression (GBR), K-Nearest Neighbors (KNN), Support Vector Machine (SVM), XGBoost, and Multi-Layer Perceptron (MLP). Tree-based ensemble models were emphasized for their effectiveness in handling mixed-type features and capturing complex non-linear relationships in polymer systems.

The optimal XGBoost model was selected and subjected to hyperparameter optimization using the Optuna framework to maximize predictive accuracy. Model performance was quantified using root mean squared error (RMSE), mean absolute error (MAE), and coefficient of determination (R^2^). RMSE measures prediction error magnitude, MAE represents average absolute deviation, and R^2^ quantifies the proportion of variance explained by the model. The RMSE, MAE, and R^2^ were calculated using Equations (1)–(3):

(1) $$RMSE=\sqrt{\frac{\sum_{i=1}^{n}{\left(y_{t}-y_{p}\right)}^{2}}{n}}$$

(2) $$MAE=\frac{\sum_{i=1}^{n}\left|y_{t}-y_{p}\right|}{n}$$

(3) $$R^{2}=1-\frac{\sum_{i=1}^{n}{\left(y_{p}-y_{t}\right)}^{2}}{\sum_{i=1}^{n}{\left(y_{t}-y_{m}\right)}^{2}}$$

where y_p_ represents the predicted output value, y_t_ denotes the reported true output value, y^m^ represents the mean of observed output values, n indicates the number of samples in the training or testing datasets. SHAP (Shapley Additive Explanations) analysis was implemented to provide interpretable insights into feature contributions and mechanistic relationships. SHAP values quantify individual feature impacts on predictions, enabling both global feature importance ranking and local explanations of formulation effects on tensile strain properties, thereby addressing the “black box” limitation of machine learning models.

## Data Availability

The data presented in this study are available on request from the corresponding authors.
